# Hypoxia induces cancer-associated cAMP/PKA signalling through HIF-mediated transcriptional control of adenylyl cyclases VI and VII

**DOI:** 10.1038/s41598-017-09549-8

**Published:** 2017-08-31

**Authors:** Veronika Simko, Filippo Iuliano, Andrea Sevcikova, Martina Labudova, Monika Barathova, Peter Radvak, Silvia Pastorekova, Jaromir Pastorek, Lucia Csaderova

**Affiliations:** 1 Institute of Virology, Biomedical Research Center, Slovak Academy of Sciences, Bratislava, Slovak Republic; 2 Department of Chemistry, Faculty of Natural Sciences, University of Ss. Cyril and Methodius, Trnava, Slovak Republic

## Abstract

Hypoxia is a phenomenon often arising in solid tumours, linked to aggressive malignancy, bad prognosis and resistance to therapy. Hypoxia-inducible factor-1 has been identified as a key mediator of cell and tissue adaptation to hypoxic conditions through transcriptional activation of many genes involved in glucose metabolism and other cancer-related processes, such as angiogenesis, cell survival and cell invasion. Cyclic adenosine 3′5′-monophosphate is one of the most ancient and evolutionarily conserved signalling molecules and the cAMP/PKA signalling pathway plays an important role in cellular adaptation to hypoxia. We have investigated possible new mechanisms behind hypoxic activation of the cAMP/PKA pathway. For the first time, we have shown that hypoxia induces transcriptional up-regulation of the system of adenylyl cyclases, enzymes responsible for cAMP production, in a panel of carcinoma cell lines of various origin. Our data prove functional relevance of the hypoxic increase of adenylyl cyclases VI and VII at least partially mediated by HIF-1 transcription factor. We have identified adenylyl cyclase VI and VII isoforms as mediators of cellular response to hypoxia, which led to the elevation of cAMP levels and enhanced PKA activity, with an impact on cell migration and pH regulation.

## Introduction

Hypoxia is a characteristic feature of a broad range of solid tumours. Activation of the hypoxic pathway, due to an inadequate supply of oxygen, is associated with aggressive malignancy, poor prognosis and resistance to therapy. Hypoxia-inducible factor-1 (HIF-1) has been identified as a master regulator of cell and tissue adaptation to hypoxic conditions through transcriptional activation of many genes involved in glucose metabolism and other cancer-related processes, such as angiogenesis, cell survival and cell invasion^1^.

Carbonic anhydrase IX (CA IX) is a HIF-1-induced metalloenzyme associated with solid tumours^2, 3^. Its activity results in the neutralisation of intracellular pH necessary for tumour cell survival and in the extracellular acidification supporting invasiveness^4, 5^. Due to its pro-survival and pro-migratory effects, CA IX is recognised as an indicator of poor prognosis with a clinical potential^6–8^. Our previous study on MDCK cells transfected with CA IX revealed that hypoxia regulates both expression and activity of CA IX^5^, and that the intact intracellular tail is critical for proper functioning of CA IX^9^. Our recent study revealed that phosphorylation of Thr443 residue in the intracellular tail by the cyclic adenosine 3′5′-monophosphate (cAMP)-dependent protein kinase A (PKA) is required for CA IX activation^10^. Increased expression and activation of PKA has been associated with cancer and linked to tumour hypoxia, which up-regulates expression of its catalytic, as well as regulatory subunits in a HIF-1 dependent manner^11^. Importantly, hypoxia can also enhance PKA activity by increasing the intracellular concentration of cAMP.

Cyclic adenosine 3′5′-monophosphate is one of the most ancient and evolutionarily conserved signalling molecules^12^. In mammals, cAMP is present in every cell type and organ where it controls a wide range of cellular processes and functions. However, cAMP signalling also participates in several pathologic processes, e.g. cancer, neurodegeneration, heart failure and diabetes^13^. Intracellular levels of cAMP are regulated by the balance between the activities of two enzyme families: adenylyl cyclases, which control cAMP production, and phosphodiesterases, which control its degradation.

cAMP is synthesised from adenosine triphosphate by adenylyl cyclases (ADCY) that are encoded by ten various genes (*ADCY* *1-10*). Nine members of them belong to the transmembrane subfamily (tm*ADCY*, encoded by *ADCY* *1-9*). They share a common structural organisation and possess a conserved catalytic mechanism^14, 15^. Cellular distribution and specific functions result from diverse mechanisms of their activation and inhibition. A pivotal role in their activation is played by heterotrimeric G proteins transducing multiple extracellular signals through G protein-coupled receptors. However, they also receive signals e.g. from PKA, protein kinase C, calmodulin, calcineurin and various small molecules. Second intracellular source of cAMP, soluble adenylyl cyclase (s*ADCY*, encoded by *ADCY* *1* *0*), has no membrane spanning motifs and is localised within the cytosol, especially in mitochondria and nucleus^16^. s*ADCY* is uniquely regulated by the intracellular bicarbonate^17^ and calcium^18^, and is insensitive to G protein-mediated regulation.

Recent research focused on cAMP brought a new perspective on its cellular distribution and associated signalling. The original model assumed cAMP generation at the plasma membrane and its diffusion through the cytoplasm in order to carry out cellular responses. Modern methods based on *in situ* measurements revealed its restricted diffusion inside signalling microdomains at the plasma membrane and other intracellular sites where cAMP is formed, degraded and localised closer to the place of action with the entire machinery of a unique composition, made up of ADCYs, phosphodiesterases (PDEs), A-kinase anchor proteins (AKAPs) and effector molecules, such as exchange proteins directly activated by cAMP (EPACs) and PKA^19–21^.

Hypoxic activation of the cAMP/PKA signalling pathway plays an important role in cell adaptation to hypoxia. As hypoxia is a phenomenon often occurring in tumours, we have studied the hypoxia-driven mechanisms behind the elevation of cAMP levels. We have especially focused on the transcriptional activation of adenylyl cyclases in hypoxia and on a possible participation of hypoxia-inducible factor-1 in this process.

## Results

### Hypoxia increases cAMP production despite higher activity of phosphodiesterases

**Figure 1:**
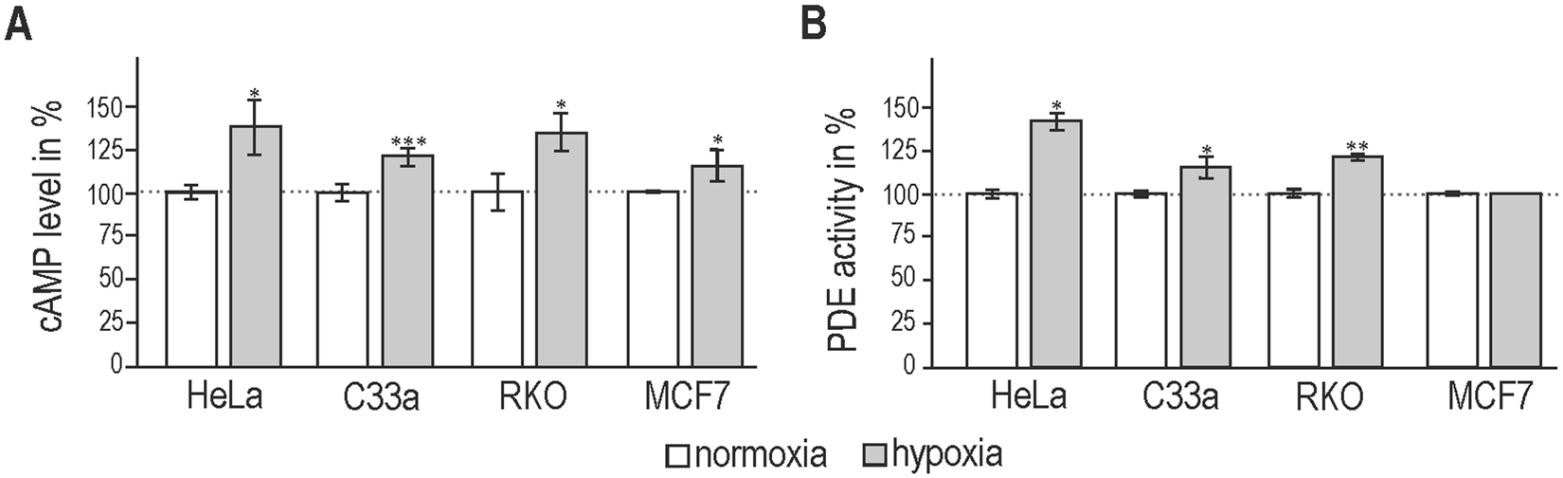
Effect of hypoxia on the level of cAMP and activity of phosphodiesterases (PDE) in different carcinoma cell lines. (**A**) Cervical carcinoma HeLa and C33a cells, colorectal carcinoma RKO cells and breast carcinoma MCF7 cells were cultured in normoxic (21% O_2_) and hypoxic conditions (2% O_2_). Following the 24 h incubation, protein extracts were prepared and level of cAMP was analysed by ELISA. cAMP levels were elevated under hypoxia in all tested cell lines. (**B**) The same protein samples were analyzed for the activity of phosphodiesterases using PDELight Assay Kit where produced bioluminiscent signal was directly proportional to PDE activity. In both assays, the results of three independent biological experiments (mean ± stdev) are expressed as % of the respective normoxic control which was set to 100%. Statistical significance of differences between normoxic and hypoxic cells was assessed using Student’s t-test (*P < 0.05, **P < 0.01, ***P < 0.001).

Our previous observation indicated an elevated cAMP concentration in canine kidney epithelial MDCK cells during hypoxia^10^. The analysis of a panel of carcinoma cell lines (HeLa, C33a, RKO, and MCF7) cultured in normoxia (21% O_2_) and hypoxia (2% O_2_, 2% H_2_, 5%CO_2_, 91% N_2_) confirmed this finding. cAMP production was significantly increased in all tested cell lines (Fig. 1A). However, duration and strength of intracellular signalling mediated through cAMP is highly affected by the action of phosphodiesterases (PDEs). These enzymes degrade cAMP and catalyze its hydrolysis to AMP^22^, thus regulating various physiological processes. As PDEs are critical down-regulators of intracellular cAMP concentrations, we determined their enzymatic activity that could be behind the hypoxic elevation of cAMP. However, three cancer cell lines showed an increased PDE activity after 24 h incubation in hypoxia, and PDE activity in MCF7 was unchanged (Fig. 1B).

### Hypoxia contributes to up-regulation of adenylyl cyclases at transcriptional level

**Figure 2:**
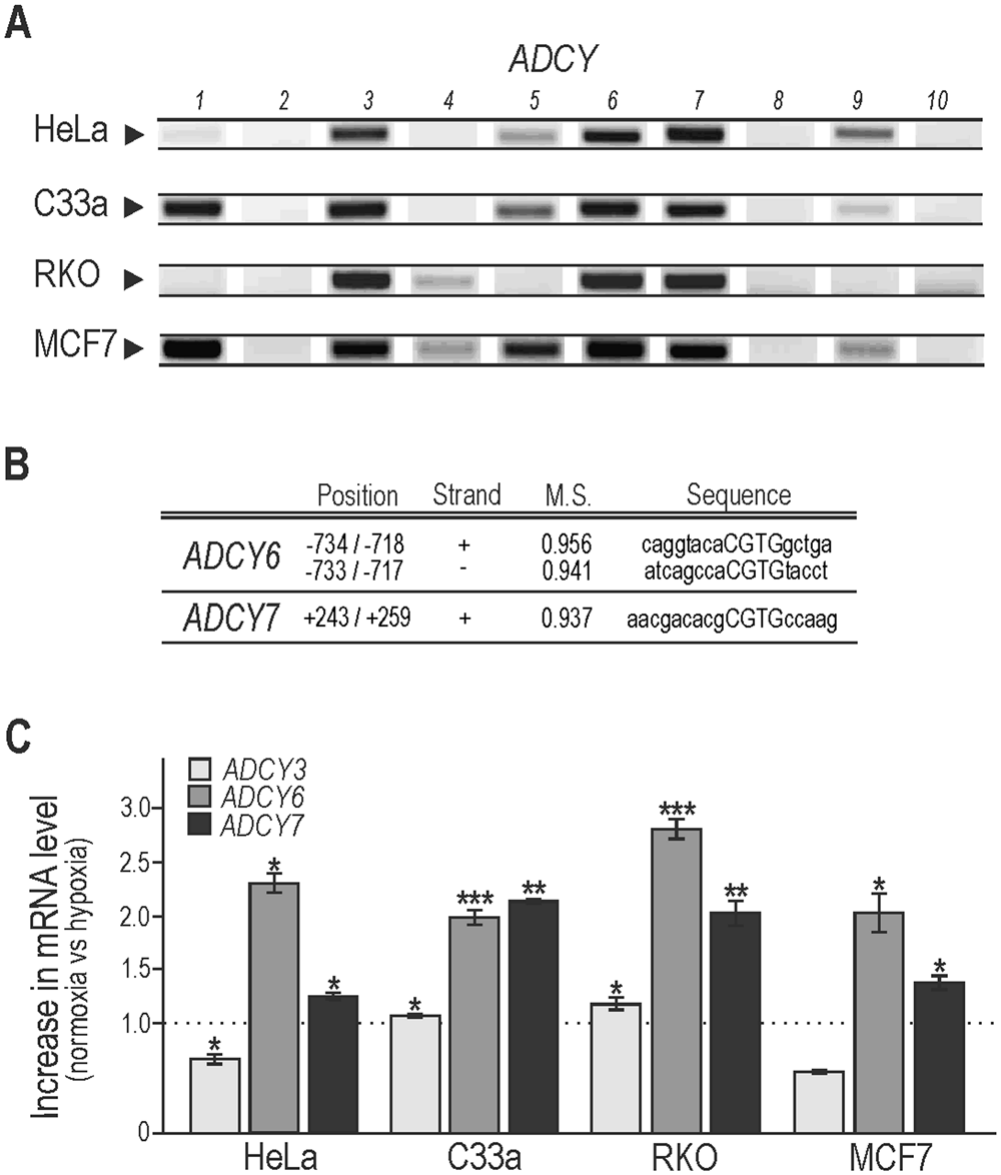
Hypoxia up-regulates transcriptional levels of adenylyl cyclases *6* and *7*. (**A**) We have analyzed the expression pattern of all ten adenylyl cyclases in hypoxia by RT PCR method. The results show moderately diverse profiles of tested carcinoma cell lines of various origin with a similar expression of isoforms *3*, *6* and *7*. Some isoforms were not detected by used PCR settings, however were amplified at higher cycles in quantitative PCR (data not shown). (**B**) Bioinformatic analysis revealed hypoxia responsive elements (HRE) with relatively high matrix similarity (M.S.) in promoter regions of *ADCY6* (transcript variant 2) and *ADCY7*. Positions of predicted HREs are localized relative to transcription start site. Capitals in column “Sequence” represent HIF-1 core binding sequences. (**C**) Four tested cell lines were cultured in normoxic and hypoxic (2% O_2_) conditions. Following 24 h incubation, the RNA was isolated, transcribed to cDNA and used in quantitative PCR. The expression of *ADCY6* and *ADCY7*, which contain HIF-1 binding site according to *in silico* analysis, was increased at mRNA level in hypoxia in all four cell lines. The mRNA level of *ADCY3*, which does not contain HIF-1 binding site, was unchanged or decreased. The graph shows changes in mRNA levels normalized to actin. The results (mean ± stdev) represent the mean from three independent biological experiments, all performed in triplicates.

In our study we have focused on investigating a possible direct transcriptional regulation of adenylyl cyclases by hypoxia in selected cancer cell lines, which could contribute to the elevation of cAMP. We analysed hypoxic mRNA profile of all ten *ADCY* genes. As expected, carcinoma cells of various origin exhibited slightly different transcriptional patterns. The most abundant isoforms present in all studied cell lines were isoforms 3, *6*, and 7, and were therefore considered further (Fig. 2A). To elucidate a link between increased cAMP and mRNA levels of some adenylyl cyclases and hypoxia, we performed detailed *in silico* promoter analysis using MatInspector software (www.genomatix.de). We focused on binding motifs of hypoxia-responsive transcription factors including HIF-1 binding sites (HRE) in the isoforms abundantly present in all our studied cell lines in hypoxia. The software predicted HIF-1 binding sites in the regulatory regions of genes encoding *ADCY6*, *ADCY7* (Fig. 2B), whereas the *ADCY3* promoter did not show any HRE element. The comparison of mRNA levels of *ADCY6* and *ADCY7* in normoxic versus hypoxic samples revealed their increased hypoxic transcription in all four cancer cell types (Fig. 2C), whereas *ADCY3* was unchanged or reduced.

### Protein levels of ADCY VI and ADCY VII are increased in hypoxia in a HIF-1 dependent manner

**Figure 3:**
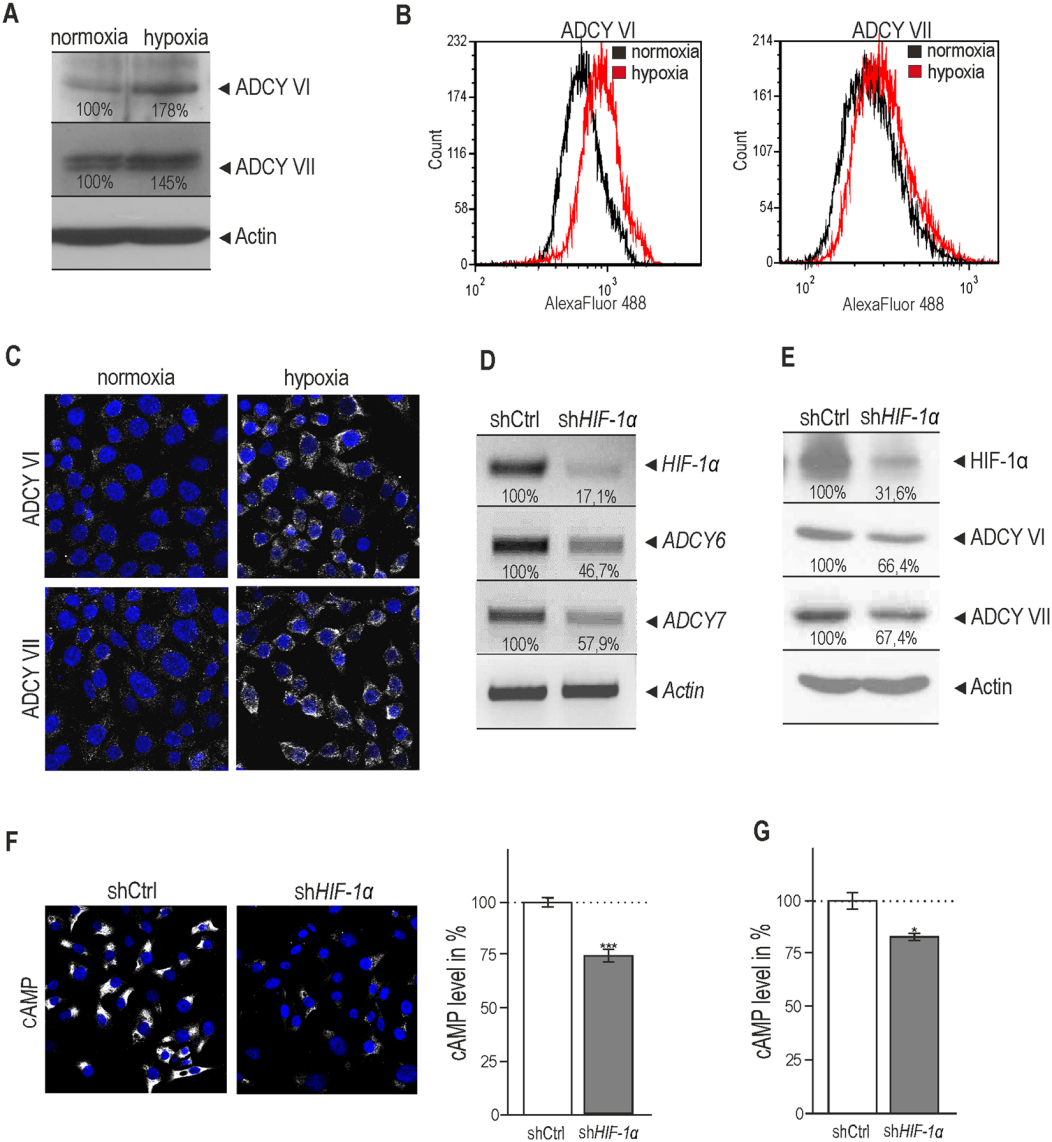
Hypoxia leads to increased protein levels of ADCY VI and ADCY VII in HIF-1α dependent manner. Protein levels of ADCY VI and ADCY VII were detected in RKO cells cultured in normoxia and hypoxia (2% O_2_) using Western blotting (**A**), flow cytometry (**B**) and immunofluorescent staining (**C**). Representative histograms (**B**) show a shift towards higher intensities of ADCY VI and ADCY VII signal in hypoxic samples. Results confirmed that isoforms VI and VII were elevated also at the protein level in hypoxia. (**D,E**) RKO cells were transiently transfected with pSuper_shHIF-1α. The following day, the transfected cells were placed into the hypoxia workstation and cultured in hypoxic conditions for 24 h. At 48 h post transfection, samples were analysed by RT PCR (**D**) and Western blotting (**E**). Suppression of HIF-1α led to reduced amount of adenylyl cyclase VI and VII. The bands were quantified in ImageJ and related to control samples (set as 100%). All results were normalized to actin. (**F**) After suppression of HIF-1α, a significantly decreased level of cAMP was observed in hypoxic RKO cells using immunofluorescent staining (left part). Graph (**F**, right part) shows the average level of cAMP signal per pixel for control and silenced samples, at least 200 cells were processed for each sample. The cAMP level in control samples was set as 100%. Results were calculated from three independent biological experiments. (**G**) To show efficiency of immunofluorescent staining performed with cAMP antibody (in **F**) we measured cAMP levels in RKO cells after suppression of HIF-1α using commercial kit (ref. 4339 Cell Signaling). Statistical significance of differences between silenced and control samples was assessed using Student’s t-test (*P < 0.05, **P < 0.01, ***P < 0.001). All images (**A–F** left part) show representative results from three independent biological experiments.

Changes in the expression of ADCY VI and ADCY VII in normoxic versus hypoxic RKO cells were proved also at protein levels by Western blotting (Fig. 3A). These results were verified by flow cytometry analysis (Fig. 3B). The histogram of analysed cells showed increased intensity of adenylyl cyclase VI and VII signal in hypoxia. Averaged geometric mean of measured intensities from three independent experiments shifted toward higher values in hypoxia: by 59.9% ± 19.9% and 35.8% ± 13.3% for ADCY VI and VII, respectively. Immunofluorescent analysis of RKO cells cultured in normoxic and hypoxic conditions (2% O_2_) for 24 h also confirmed a significant increase at the protein level of both ADCY VI and ADCY VII isoforms (Fig. 3C). Average intensity of immunofluorescent signal from three independent experiments per pixel increased in hypoxia by 10.1 ± 4.1% and 11.5 ± 4.7% for ADCY VI and VII. As *in silico* analysis predicted the involvement of HIF-1 transcription factor in the hypoxic elevation of the ADCY isoforms amounts, we used transient silencing of the *HIF-1α* subunit to verify a possible role of HIF-1 in this process. Results showed reduced mRNA (Fig. 3D) and protein levels of ADCY VI and ADCY VII in hypoxic RKO cells after *HIF-1*α silencing (Fig. 3E). The suppression of HIF-1α in RKO cells also considerably reduced the concentration of cAMP in hypoxia as detected by immunofluorescent staining (Fig. 3F) and also verified by ELISA (Fig. 3G). Obtained data support the role for HIF-1 as a possible transcriptional regulator of these *ADCYs* during hypoxia. Thus, it appears that hypoxia can control an important step of cAMP generation through HIF-1α also at the level of adenylyl cyclases expression.

### Suppression of *ADCY6* and *ADCY7* during hypoxia leads to down-regulation of cAMP/PKA signalling

**Figure 4:**
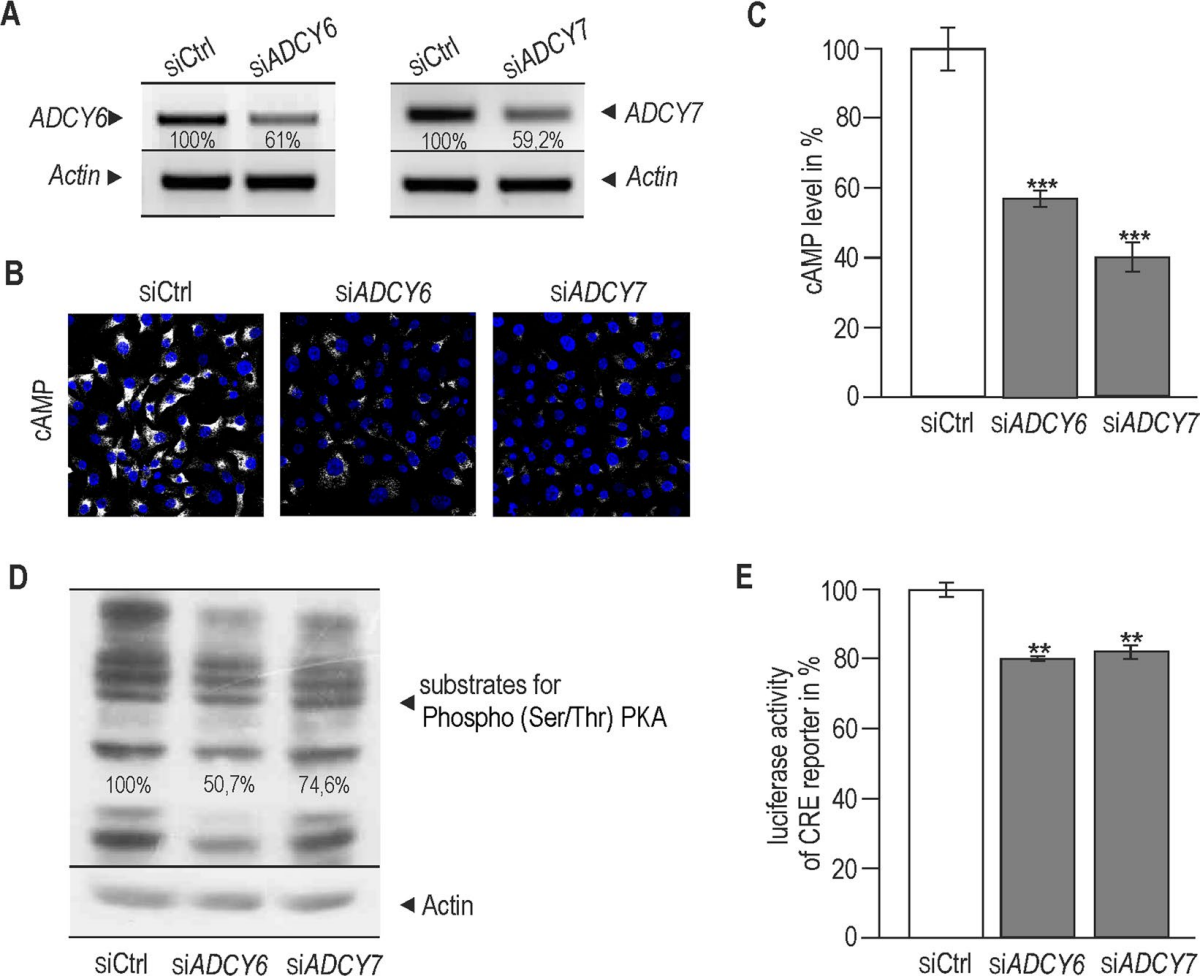
Suppression of *ADCY6* and *ADCY7* leads to decrease of cAMP level and reduced cAMP/PKA signalling. RKO cells were transiently transfected with specific *ADCY6* and *ADCY7* siRNA. The following day transfected cells were placed in the hypoxia station and cultured in hypoxic conditions (2% O_2_) for 24 h. At 48 h post transfection, samples were verified by semiquantitative PCR (**A**). The bands were quantified in ImageJ and related to control samples (set as 100%). All results were normalized to actin (**A**). After suppression of *ADCY6* and *ADCY7* a significantly decreased level of cAMP was observed in hypoxic RKO cells using immunofluorescent staining (**B**). cAMP is shown as a greyscale signal where a shift from grey to white represents an increase in the signal intensity. Images were acquired at LSM Meta 510 confocal microscope (Zeiss) at the same settings. Graph (**C**) shows a change in the average intensity of cAMP signal per pixel (measured in levels of greyscale) in silenced samples compared to controls. At least 200 cells were analyzed for each sample. Results were calculated from three independent biological experiments and expressed as cAMP level in % of the control samples levels set as 100%. Statistical significance of differences between silenced and control samples was assessed using Student’s t-test (*P < 0.05, **P < 0.01, ***P < 0.001). Moreover, silencing of *ADCY6* or *ADCY7* led to a decreased level of substrates for Phospho (Ser/Thr) PKA analyzed by Western blotting (**D**). All bands of substrates for Phospho (Ser/Thr) PKA shown in Western blot were quantified in ImageJ, the control sample was set as 100% and results were normalized to actin. (**E**) The effect of these isoforms on cAMP signalling was confirmed by a significant reduction of CRE reporter activity in samples with silenced *ADCY6* and *ADCY7*, measured by dual luciferase assay (Promega). Graph shows the mean of two independent experiments, each performed in quadruplicates. Results showed in **A,B** and **D** give representative images from three independent biological experiments.

Transient silencing of *ADCY6* and *ADCY7* was used to assess their contribution to the hypoxic elevation of cAMP (Fig. 4A). After their suppression, we detected a significant reduction of cAMP signal (Fig. 4B,C). Similarly, levels of phospho-substrates of PKA were decreased in hypoxia after silencing of both *ADCY* isoforms (Fig. 4D). These data prove functional consequences of suppressing *ADCY6* and *ADCY7* for PKA activity in hypoxia. Reduction in the phosphorylation status of PKA substrates was considerably higher when RKO cells were subjected to shRNA targeting of *HIF-1α* (data not shown). This is in agreement with the previous evidence denoting HIF-1 as a regulator of hypoxic activation and expression of PKA^11^. The effect of *ADCY6* and *ADCY7* suppression on cAMP signalling during hypoxia was confirmed by luciferase assay in which the activity of CRE reporter was reduced significantly by almost 20% (Fig. 4E).

### Suppression of *ADCY6* and *ADCY7* affects migration capacity of hypoxic cells

**Figure 5:**
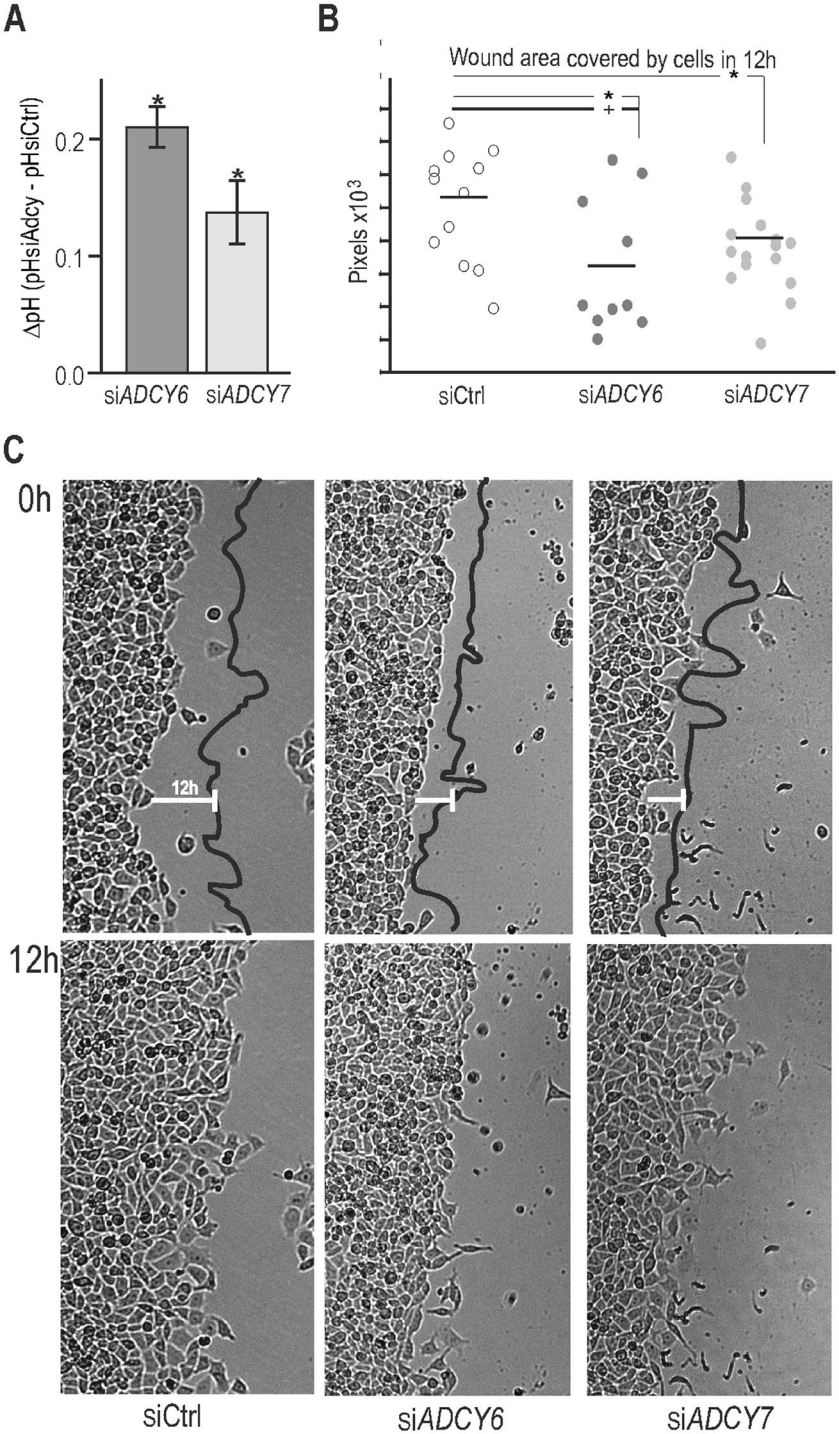
Suppression of *ADCY6* and *ADCY7* slows migration capacity of hypoxic RKO cells. (**A**) The graph shows a reduction in the extracellular acidification, i.e. increase in pH_e_, of hypoxic RKO cells with silenced *ADCY6* and *ADCY7* (compared to scramble control) after 24 h culture in hypoxia (2% O_2_), 48 h post transfection. Data represent the mean ± stdev of three independent experiments. Acidification of extracellular space reflects the enzymatic activity of NHE1 and CA IX (hypoxia-induced, tumour-associated protein) which are also involved in the cell migration process. (**B**) The graph depicts the results of the wound healing assay given as the area covered by cells, migrating to close the wound, at 12 h after the scratch, measured at various positions along the wounds. Cells with silenced *ADCY6* or *ADCY7* exhibited slower migration which is clearly visible in representative graph (**B**) and images (**C**) of the wound healing assay. The assay was repeated three times. (**C**) Images were taken at the start of the wound healing assay (0 h) and 12 h after the scratch. Statistical significance of differences between silenced and control samples was assessed using Student’s t-test (*P < 0.05, **P < 0.01, ***P < 0.001). Because of multiple comparisons, we also performed ANOVA with Dunnet’s post-hoc test, the difference between scramble control (siCtrl) and silenced *ADCY6* remained significant at P < 0.05 (+).

There is mounting evidence that PKA is an important regulator of cell migration through phosphorylation and activation of several components of the pH-regulating machinery^11, 23^. These include Na^+^/H^+^ exchanger 1^24, 25^ and CA IX^10, 26^ which contribute to intracellular alkalinisation and extracellular acidification at the leading front area of migrating cells. In agreement with this evidence, we found that silencing of *ADCY6* and *ADCY7* genes resulted in reduced acidification of the culture medium of hypoxic RKO cells when compared to scramble control (∆pH~0.2, Fig. 5A). This indicated a possible contribution of hypoxia-up-regulated *ADCY* isoforms in PKA activation. To further assess functional relevance of thus affected PKA activation we performed a wound healing assay. Results showed a decreased migration rate of RKO cells after suppression of *ADCY6* and *ADCY7* (Fig. 5B,C) supporting our assumptions. The migration was slowed to 74.2 ± 8.8% and 82.9 ± 6.4% of the control samples after silencing of *ADCY* *6* and *7*, respectively (mean ± stdev of three independent experiments).

## Discussion

Cyclic adenosine 3′5′-monophosphate is one of the most ancient and evolutionarily conserved signalling molecules responding to various stimuli and controlling diverse range of cellular processes. Different receptors are responsible for cAMP accumulation in cells that causes different physiological outcomes. In humans, the cAMP synthesis from ATP is mediated by nine transmembrane adenylyl cyclases and one soluble isoform. In hypoxia, the cAMP/PKA signalling pathway plays an important role in cells adaptation to oxygen insufficiency. During hypoxia, changes in cAMP levels increase PKA activation and enhanced expression of PKA subunits occurs. PKA directly contributes to tumour biology, particularly to hypoxia-mediated invasion and migration^11^. Moreover, PKA directly cooperates with a key regulator of molecular responses to hypoxia. Bullen *et al*. demonstrated that PKA phosphorylated Thr63 and Ser692 on HIF-1α and thus inhibited its proteosomal degradation in HeLa carcinoma cells and rat cardiomyocytes^27^. PKA also stimulated the binding of the coactivator p300 to HIF-1α and enhanced HIF-1α transcriptional activity and target gene expression. In this manner, PKA influences a panel of molecules induced by hypoxia, some of them also *via* direct phosphorylation (e.g. Thr443 of carbonic anhydrase IX^10^). Furthermore, increased cAMP concentrations enhanced the expression of HIF target gene encoding CD73, an enzyme that converts extracellular adenosine 5′-triphosphate to adenosine^28, 29^. Release and extracellular accumulation of adenosine is one of the many adaptive mechanisms that have evolved as a protective response to hypoxia^30, 31^. This molecule participates in the optimal functioning of hypoxic tissues and mediates numerous metabolic effects^32^. Moreover, hypoxia also increases expression of A2A and A2B subtypes of adenosine receptors^33, 34^. In this manner adenosine signal can be transmitted through G stimulatory proteins and enhance activation of adenylyl cyclases, enzymes responsible for cAMP generation.

In our study, we have proved hypoxia-mediated increase in cAMP levels in various cancer cells *in vitro* (Fig. 1A). However, it is known that duration and strength of intracellular signalling mediated through cAMP is highly affected by the action of phosphodiesterases (PDEs). Eleven PDE families have been identified, among them cAMP specific isoforms (PDE 4, 7, 8) or members with dual specificity also for cyclic guanosine monophosphate (PDE 1, 2, 3, 10, 11). PDEs degrade cAMP and catalyze its hydrolysis to AMP^22^, thus regulating various physiological processes. Changes in their expression or enzyme activity have been associated with various pathologies including cancer^35^. As PDEs are critical down-regulators of intracellular cAMP concentrations we also determined their enzyme activity that could be behind hypoxic elevation of cAMP. Our results showed an increased or unchanged PDE activity after 24 h incubation in hypoxia in various cancer cell lines (Fig. 1B). In fact, this observation was in line with the recent report that hypoxia can induce expression of PDE 4 isoform^36^. Therefore, phosphodiesterase activity cannot be behind the mechanism responsible for elevated levels of cAMP in hypoxia. Moreover, intracellular cAMP levels may also be affected by its efflux into the extracellular space mediated by multidrug resistance proteins belonging to the ABC transporter superfamily^37, 38^. However, measurement of cAMP in culture medium from normoxic versus hypoxic HeLa and C33a cells using ELISA method revealed no significant changes in either cell line (data not shown).

Further, we have focused on the transcriptional activation of adenylyl cyclases in hypoxia and on its possible participation in hypoxic activation of the cAMP/PKA pathway. For the first time, we showed that hypoxia induces transcriptional up-regulation of adenylyl cyclases 6 and 7 in a panel of carcinoma cell lines of various origin (Fig. 2C). Our data prove functional relevance of hypoxic increase of adenylyl cyclases VI and VII at least partially mediated by the HIF-1 transcription factor (Fig. 3D,E). HIF-1α is a key transcription factor controlling responses to hypoxia. In addition, various transcription factors e.g. SP1/SP3, NFκB, CREB, AP1, p53, Egr1, Ets, Smad *etc*. are also induced in hypoxia and have been demonstrated to be hypoxia-responsive^39^. All together they contribute to the transcriptional machinery activated by this important pathophysiological stimulus supporting processes such as tumour development. Due to the increased cAMP levels (Fig. 1A) and mRNA and protein levels of adenylyl cyclases VI and VII (Figs 2C, and 3A,B,C) as a consequence of hypoxia, we have performed *in silico* promoter analysis using MatInspector software. This analysis predicted potential HIF-1 binding sites in the regulatory regions of both *ADCY6* and *ADCY7* genes *(*Fig. 2B). Moreover, analysis showed also abundant occurrence of above-mentioned hypoxia-associated transcription factor binding elements, located upstream as well as downstream to the transcription start sites.

To date, only *ADCY7* has been linked to hypoxic up-regulation at both transcriptional and protein levels in a mouse model of hypoxia-induced retinopathy^40^. We used Genevestigator tool (https://genevestigator.com/gv/) to analyze the expression of adenylyl cyclases comparing normal and hypoxic samples. Only a small number of Genevestigator datasets is available for such search. They revealed that only *ADCY7* out of all ten isoforms was detected as an up-regulated gene in two independent hypoxic studies. Both breast and pancreatic carcinoma cells showed two-fold elevation in hypoxic samples compared to normoxia. In both studies *ADCY7* correlated with HIF-1-induced tumor-associated carbonic anhydrase 9.

**Figure 6:**
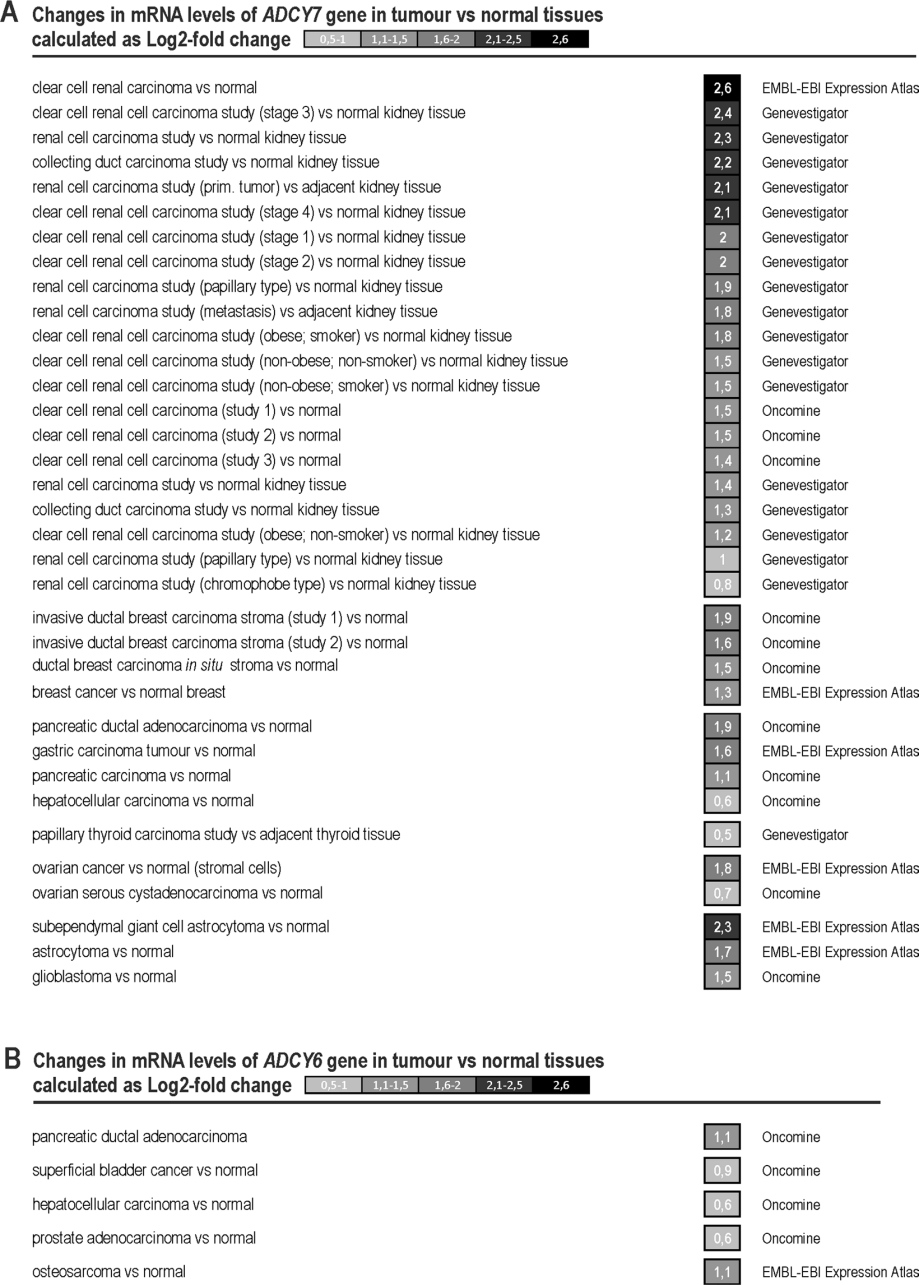
Changes in mRNA levels of *ADCY7* and *ADCY6* genes in human carcinoma tissues versus normal tissues according to data analysed from Genevestigator, EMBL-EBI Expression Atlas and Oncomine databases.

Moreover, comparison of tumour versus normal tissues samples across Genevestigator, Oncomine (www.oncomine.org) and Expression Atlas (http://www.ebi.ac.uk/) databases showed up-regulation of the *ADCY7* gene mostly in renal, breast, pancreas, gastric, ovarian cancer and hepatocellular carcinoma in several microarray and RNA-sequencing studies (Fig. 6A). Recent research also indicates differences between 2D and 3D cell cultures. Databases work with clinical samples which may represent the tumour environment which is different from hypoxic 2D culture. All these reasons could be behind difficulties in detecting *ADCY*6 up-regulation in real tumour samples although there are couple of studies showing up-regulation of *ADCY6* in tumour compared to normal tissues (*e.g*. osteosarcoma, prostate adenocarcinoma, pancreatic ductal adenocarcinoma and bladder cancer) (Fig. 6B).

Our findings support the view that hypoxia induces the cAMP signalling pathway at several upstream and downstream levels. We suggest that up-regulation of adenylyl cyclases participates in the hypoxia-induced elevation of intracellular cAMP. In these processes HIF-1 seems to play an important role and to contribute also to an increased expression of isoforms ADCY VI and ADCY VII, resulting in higher cAMP generation that affects PKA function, with impact on cell migration and pH regulation.

cAMP represents a molecule of interest which also plays a role in the process of tumorigenesis, particularly by being a part of tumour cells adaptation to reduced oxygen levels reported in carcinoma.

## Materials and methods

### Cell culture

HeLa and C33a cervical carcinoma cells, RKO colorectal carcinoma cells and MCF7 breast carcinoma cells were cultured under standard conditions in Dulbecco’s modified Eagle’s medium supplemented with 10% foetal calf serum (Bio Whittaker) and gentamicine (Sandoz) in humidified air containing 21% O_2_, 5% CO_2_ at 37 °C and in hypoxic conditions at anaerobic workstation (2% O_2_, 2% H_2_, 5%CO_2_, 91% N_2_, Ruskinn Technology). All cell lines were purchased from ATCC and regularly tested for Mycoplasma contamination.

### cAMP assay

Cell lysates were prepared using RIPA lysis buffer (1% Triton X-100 and 1% deoxycholate in PBS) and diluted in PBS to concentration 0.5 μg/μl. 100 μl of protein samples were loaded onto 96 well plate (high binding microlon 600, ref. 655061, Greiner bio-one) in triplicates and incubated at 37 °C overnight. The following day samples were washed with 0.02% Tween-20 in PBS, blocked with 10% FCS in wash buffer for 1 h, incubated with anti-cAMP antibody (1 μg/ml) for 3 h, washed again and incubated with HRP-conjugated secondary antibody for 1 h. All steps were performed at room temperature. Absorbance and colour changes after development with orthophenylene diamine were measured at 492 nm. Three independent biological experiments were performed, each in triplicates. Alternatively, detection of cAMP level was performed with commercial kit (ref. 4339 Cell Signaling) according to manufacturer’s instructions. Two independent biological experiments were performed, each in triplicates.

### PDE activity assay

Activity of phosphodiesterases was determined using the PDELight Assay kit (Lonza). 20 μl of cell lysates prepared using RIPA lysis buffer were analysed according to the manufacturer’s instructions. Three independent biological experiments were performed, each in triplicates.

### Transient transfection

The cells were plated onto 30-mm Petri dishes to reach approximately 70% monolayer density on the following day. Transfection was performed with 2 μg of the plasmid (pSuper_shCtrl, pSuper_shHIF-1α) or with 25 nM siRNA (Thermo Scientific, non-targeting control pool D-001810-10, On-TARGETplus SMART pool human *ADCY6*_L-006636-00 and *ADCY7*_L-006801-00) using 2 μl Attractene transfection reagent (Qiagen). For luciferase reporter assay, transfection was performed with 1 μg of the luciferase reporter plasmid containing cAMP response elements (Affymetrix), 50 ng of pRL-TK renilla vector (Promega) and 25 nM siRNA using 2,5 μl Attractene transfection reagent according to the manufacturer’s recommendations. After 24 h, the transfected cells were trypsinised, plated according to the type of further experiment, allowed to attach and cultured in normoxia and hypoxia for 24 h.

### RT PCR and quantitative (q) PCR

Total RNA was isolated using Trizol solution (Sigma) and reverse transcription of 3 μg RNA for each sample was performed with the High-Capacity cDNA Reverse Transcription kit (Applied Biosystems) according to the manufacturer’s recommendations. Semiquantitative RT PCR was carried out using Dream Taq Green PCR Master mix (Thermo Scientific). Each PCR reaction contained 5 μl of 10x diluted cDNA and ran for 3 min at 95 °C for initial denaturation followed by 30 cycles of 95 °C-60 °C-72 °C (all steps for 30 s) and final elongation at 72 °C for 5 min. Q-PCR was carried out using Maxima Syber Green PCR Master mix (Thermo Scientific) and ran for 10 min at 95 °C for initial denaturation followed by 40 cycles of 95 °C for 15 s and 60 °C for 1 min. Sample Ct values were normalised to actin. Relative expression was calculated using the ΔΔCt method. All amplifications were performed in triplicate. Results were calculated from three independent experiments. The bands for RT PCR were quantified in ImageJ software. All oligonucleotides used for RT PCR and q-PCR are as follows: *ADCY1*_S: 5′ccttttggtcaccttcgtgt3′, *ADCY1*_A: 5′ctcagtcagaatccgcacaa3′, *ADCY2*_S: 5′aacagcacctggtgaaaacc3′, *ADCY2*_A: 5′tgatcttgccgttctctgtg3′, *ADCY*3_S: 5′cattggcagcacgtatatgg3′, *ADCY3*_A: 5′gcgcagcatgaagttattga3′, *ADCY4*_S: 5′actgctgatgacccgttacc3′, *ADCY4*_A: 5′ccactgtttctgcgagttga3′, *ADCY5*_S: 5′ttcttcaacaacgggacctc3′, *ADCY5*_A: 5′cctgcagtttccagaggaag3′, *ADCY*6_S: 5′ggcagctggaaaagatcaag3′, *ADCY6*_A: 5′gcccaatcttcatctggaaa3′, *ADCY*7_S: 5′gagatgctgtcagccattga3′, *ADCY7*_A: 5′agcaggatgcagacgaagat3´, *ADCY8*_S: 5′caggtcatcctccaagtggt3′, *ADCY8*_A: 5′cgctcctgtctttggttctc3′, *ADCY9*_S: 5′cgctcaaggtctgaacttcc3′, *ADCY9*_A: 5′cggggtcaccagtacctaga3′, *ADCY10*_S: 5′cgttttccaacaaacggagt3′, *ADCY10*_A: 5′cgttggaggtgacaggtttt3′, *HIF-1α*_S: 5′gcttggtgctgatttgtgaacc3′, *HIF-1α*_A: 5´gcatcctgtactgtcctgtggtg3′, *Actin*_S: 5′ccaaccgcgagaagatgacc3′, *Actin*_A: 5′gatcttcatgaggtagtcagt3′.

### Western blotting

Cells grown in confluent monolayers were rinsed twice with PBS, resuspended in ice-cold lysis buffer (1% Triton X-100; 50 mM Tris pH7.5; 150 mM NaCl; 0.5% Nonidet P-40) containing protease (Roche) and phosphatase inhibitors cocktail (Sigma Aldrich), disrupted by sonication and cleared by centrifugation. Protein concentrations were quantified using the BCA protein assay reagents (Pierce). The extracts (100 μg/lane) were resolved in 8% SDS-PAGE and transferred to a PVDF membrane (Macherey-Nagel). Protein bands were visualised using an enhanced chemiluminescence kit (GE Healthcare Bio-Sciences) and quantified in ImageJ software (using Analyze-Gels). All results were normalised to actin.

### Immunofluorescence

RKO cells grown on glass coverslips for 24 h in hypoxia were fixed in 4% paraformaldehyde at room temperature for 20 min and permeabilised with 0.02% Tween-20 in PBS. After blocking in 3% BSA in PBS for 1 h, the cells were incubated with primary antibody against cAMP, ADCY VI or ADCY VII overnight at 4 °C. The following day, the cells were washed four times for 10 min with 0.02% Tween-20 in PBS, incubated for 1 h at 37 °C with Alexa-conjugated secondary antibody, and washed three times with PBS. Finally, the cells were mounted in Fluoroshield Mounting Medium with DAPI (Abcam) and analysed by laser scanning microscopy (LSM 510 Meta Microscope; Zeiss, objective 40x). The staining of all samples was performed under the same conditions. All immunofluorescent images were acquired at the same microscope settings for all compared samples.

### Quantification of cAMP fluorescent signal

Images of cells with fluorescent cAMP signal were processed in the ImageJ software. Intensity of grayscale images reflected cAMP amount, the brighter pixels meaning higher levels of cAMP. Images were thresholded according to their intensity to exclude pixels with background only. Only pixels with the cAMP signals were analysed, and the average intensity per pixel was calculated for cAMP signal for normoxic and hypoxic cells, or cells with silenced *HIF-1α*, *ADCY6* or *ADCY7*. At least 200 cells were processed for each sample. Results were calculated from three independent biological experiments.

### Flow cytometry analysis (FACS)

RKO cells were cultured under normoxic and hypoxic conditions. After 24 h, the cells were detached using trypsin, resuspended in 1% FCS in PBS. After centrifugation, the cells were fixed in ice-cold 70% ethanol for 30 min at 4 °C, centrifuged and washed twice with 1% FCS in PBS. For measurement of the expression of ADCY VI and ADCY VII, the cells were incubated with primary antibodies overnight at 4 °C. After incubation, the cells were centrifuged, washed twice and incubated with secondary antibody for 1 h at 4 °C, and then washed four times. Cells stained with secondary antibody only were used as negative control. The samples were analysed using a Guava EasyCyte Plus flow cytometer (Millipore) with FCS Express software. The staining of all samples was performed under the same conditions. Geometric mean of measured intensities was calculated from three independent experiments.

### pH measurement

pH of cell culture media was measured by a special microelectrode (Mettler Toledo, InLab® Micro) designed for measurement in small volumes. The measurement by the electrode was done directly in the hypoxic workstation under the oxygen concentration of 2%. The graph in Fig. 5A gives the difference between pH of the culture medium of cells with silenced ADCY isoforms and medium of control cells. Data are presented as mean ± stdev of three independent experiments.

### Wound healing assay

RKO cells transfected with si*ADCY6*, si*ADCY7* and non-targeting siRNA control (siCtrl), were seeded to confluence on tissue culture plates 24 h post transfection. Cells were left to attach, moved into hypoxic workstation and cultured for 24 h in 2% O_2_. As a next step, the cells were starved in DMEM with 0.5% FCS for 6 h in 2% O_2_. A wound was made with a sterile micropipette tip. Floating cells were removed by washing with PBS. Fresh DMEM with 0.5% FCS and hepatocyte growth factor (HGF, 5 ng/ml, Sigma) was then added. Time-lapse acquisition was performed at Zeiss Cell Observer System at magnification 100x, in the incubation chamber at 37 °C in 2% O_2_ and 5% CO_2_ atmosphere. Imaging was managed by Axiovision 4.8 software, using the Multidimensional Acquisition settings. We evaluated the movement of the migrating fronts of cells covering the wound for at least 10 positions (for each sample). Positions were selected at a sufficient distance from each other and their fields of view never overlapped. Wound healing was quantified using ImageJ software as the wound area covered by cells in 12 h and results were compared by t-test. The assay was repeated three times.

### Antibodies

Primary antibodies: mouse monoclonal anti-human HIF-1α (1:250, ref. 610959 BD Transduction Laboratories); goat polyclonal anti-human actin (1:1000, ref. sc1615 Santa Cruz Biotechnology); in house mouse monoclonal anti-human carbonic anhydrase IX- M75 hybridoma medium (1:3); rabbit polyclonal anti-human ADCY VI (1:250 in WB, ref. SAB2100054 Sigma); rabbit polyclonal anti-human ADCY VI (1:50 in IF and FACS, ref. sc25500 Santa Cruz Biotechnology); goat polyclonal anti-human ADCY VII (1:500 in WB, ref. sc32120 Santa Cruz Biotechnology), rabbit polyclonal anti-human ADCY VII (1:50 in IF and FACS, ref. SAB1303163 Sigma); rabbit monoclonal anti-human phospho-PKA substrates (1:1000, ref. 100G7E Cell Signaling); mouse monoclonal anti- cAMP (ref. ab24851 Abcam). Secondary antibodies: Alexa Fluor 488-conjugated donkey anti-mouse IgG; Alexa Fluor 488-conjugated donkey anti-rabbit IgG; (both 1:1000, Invitrogen); HRP-conjugated goat anti-mouse IgG (1:5000 in Western blotting, 1:2000 in ELISA, Dako); HRP-conjugated rabbit anti-goat IgG (1:5000, Dako); HRP-conjugated goat anti-rabbit IgG (1:5000, Dako).
